# Highly Efficient Ultraviolet Third‐Harmonic Generation in an Isolated Thin Si Meta‐Structure

**DOI:** 10.1002/advs.202404094

**Published:** 2024-07-08

**Authors:** Yanhui Deng, Zhonghong Shi, Yaqin Zheng, Houjiao Zhang, Haoyang Li, Siyang Li, Zhang‐Kai Zhou

**Affiliations:** ^1^ State Key Laboratory of Optoelectronic Materials and Technologies School of Physics Sun Yat‐sen University Guangzhou 510275 China

**Keywords:** confined hybrid anapole mode, electric field enhancement, nonlinear optics, slotted silicon disk‐ring, third‐harmonic generation

## Abstract

Nonlinear nanophotonic devices have shown great potential for on‐chip information processing, quantum source, 3D microfabrication, greatly promoting the developments of integrated optics, quantum science, nanoscience and technologies, etc. To promote the applications of nonlinear nanodevices, improving the nonlinear efficiency, expanding the spectra region of nonlinear response and reducing device thickness are three key issues. Herein, this study focuses on the nonlinear effect of third‐harmonic generation (THG), and present a thin Si meta‐sructure to improve the THG efficiency in the ultraviolet (UV) region. The measured THG efficiency is up to 10^−5^ at an emission wavelength of 309 nm. Also, the THG nanosystem is only 100 nm in thickness, which is two–five times thinner than previous all‐dielectric nanosystems applied in THG studies. These findings not only present a powerful thin meta‐structure with highly efficient THG emission in UV region, but also provide a constructive avenue for further understanding the light–matter interactions at subwavelength scales, guiding the design and fabricating of advanced photonic devices in future.

## Graphical abstract

By utilizing the confined hybrid anapole mode, the ultra‐thin silicon‐based meta‐structure can not only achieve ultraviolet third harmonic generation (THG), but also greatly enhance the conversion efficiency to as high as 10^−5^, which is a new record to date of the ultraviolet‐region THG in the all‐dielectric nanosystems.

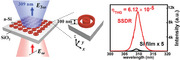

## 1 Introduction

With the rapid growing of the nanotechnology, various strategies for controlling light at nanoscale have been demonstrated, leading to large amounts of advanced compact nanophotonic devices and chips,^[^ ^1^, ^2^, ^3^, ^4^ ^]^ which greatly improve the function stability, size miniaturization and processing speed of optical and optoelectronic systems. Specifically, due to the progresses achieved in the fields of nanophotonics and material science, nanostructures with large optical nonlinearity have been widely introduced, such as 2D materials,^[^ ^5^, ^6^ ^]^ perovskites,^[^ ^7^ ^]^ semiconductor materials,^[^ ^8^ ^]^ and artificial meta‐structures,^[^ ^9^, ^10^ ^]^ giving rise to remarkable applications of optical encoding,^[^ ^11^ ^]^ quantum source and imaging,^[^ ^3^, ^12^, ^13^ ^]^ as well as biochemical monitoring,^[^ ^14^ ^]^ notably facilitating the developments of integrated optics, quantum science, biomedical engineering, pharmaceutical chemistry, etc.^[^ ^15^, ^16^ ^]^

Nonlinear harmonic generation, such as the second‐ and third‐harmonic generation (SHG and THG), is an important and extensively‐applied nonlinear phenomenon.^[^ ^17^, ^18^, ^19^, ^20^, ^21^ ^]^ It is a frequency conversion process that can upconvert optical signals into the visible or ultraviolet (UV) range via multiple waves mixing, and has a wide range of applications in the field of new optical devices such as laser sources for optical communications, biomolecular detection, and sensors.^[^ ^22^, ^23^, ^24^ ^]^ Despite of the significant achievements, there are actually several problems which may hinder the further developments of nanophotonic devices based on the nonlinear harmonic generation.

The first problem is the limited spectral range of emission light. Normally, there are two kinds of nanosystems applied for nonlinear harmonic generation, which are the plasmonic and all‐dielectric systems (we can classify the plasmonic/dielectric hybrid system as the plasmonic type). Benefiting from the large local electric field caused by the plasmon resonances, a large number of plasmonic nanostructures have been proposed, but the emission wavelengths of the nonlinear conversion achieved by the plasmonic are mainly concentrated in the range of 400–600 nm.^[^ ^25^, ^26^, ^27^ ^]^ On the other side, the all‐dielectric nanostructures with advantages of high damage threshold and low‐loss have gain much research efforts, but still, in the majority of previous works the emission wavelengths are between 400 and 800 nm.^[^ ^28^, ^29^, ^30^ ^]^ For example, Yuri S. Kivshar et al. achieved a THG emission at a wavelength of 420 nm based on silicon nanodisks. Also, David Hähnel et al. reported a THG of 517 nm in silicon metasurfaces. Despite of these progresses, it is found that there is a demand to expand nonlinear wavelengths toward shorter wavelengths driven by UV coherent light sources with better beam quality, higher energy, and higher processing accuracy.^[^ ^31^ ^]^

Secondly, it is also highly desired to find approaches for improving the nonlinear efficiency of harmonic generation. Although there is a continuing effort to improve the conversion efficiency of harmonic signals based on various photonics modes including the electric and magnetic Mie‐type modes,^[^ ^32^, ^33^, ^34^ ^]^ quasi bound states in the continuum mode^[^ ^35^, ^36^, ^37^, ^38^ ^]^ and anapole modes, the THG conversion efficiency (*η*_THG_) is still relatively low, especially in the UV band below 400 nm. For example, the *η*_THG_ at wavelengths of 313 nm, 300 nm and 280 nm are 4 × 10^−8^,^[^ ^33^ ^]^ 5.5 × 10^−8[^ ^34^ ^]^ and 5.2 × 10^−8^,^[^ ^38^ ^]^ respectively. So, to increase the *η*_THG_ in the UV band is in special need. Thirdly, it is found that, reducing device thickness may be helpful in lowering costs and losses,^[^ ^11^ ^]^ device miniaturization,^[^ ^39^ ^]^ as well as boosting 3D stacking of ultrathin chip packages.^[^ ^40^ ^]^ So, from the perspective of on‐chip integration, it would be preferred to minimize the thickness of nonlinear devices. Therefore, a solution for achieving efficient UV harmonic generation based on thin meta‐structures is urgently in need.

In order to achieve the task, we propose the slotted Si disk‐ring (SSDR) meta‐structure with confined hybrid anapole (CHA) mode for producing efficient THG. Due to the constructive coherence of the anapole and magnetic quadrupole (MQ) modes, the CHA mode can induce both small radiative dissipation and strong electric field (EF) enhancements. Therefore, by engineering the CHA mode of the SSDR meta‐structure with thickness of only 100 nm, we experimentally demonstrate a high THG conversion efficiency *η*_THG_ up to 6.12 × 10^−5^ at emission wavelength of 309 nm, which not only expands the emission spectral range of nonlinear harmonic generation, but also creates a new *η*_THG_ record about THG conversion efficiency in the UV band.^[^ ^36^, ^41^ ^]^ The demonstrated high‐energy signal of UV THG and efficient conversion efficiency in our thin meta‐structure offer promising prospects for building advanced and integrated nonlinear meta‐devices in future.

## 2 Results

### 2.1 A Two‐Step Method to Constructure the Confined Hybrid Anapole Mode

We focus on the THG system to expand the emission range of harmonic generation according to two considerations. For one thing, comparing with the SHG, the THG process can be induced more easily since its symmetry‐induced selection rules are more relaxed; furthermore, its emission wavelength is one third of the excitation wavelength, making it be more suitable for generating emission in the high‐energy region (i.e., the UV or more shorter wavelength region). Another reason is that, comparing with higher‐order harmonic generation, it is easier to obtain a high conversion efficiency in THG process because of the relatively larger third‐order nonlinear susceptibility (*χ* ^(3)^).

**Figure 1:**
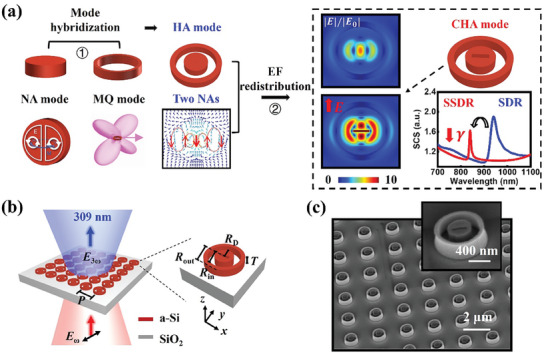
The design for CHA mode of the isolated SSDR meta‐structure. a) Illustration of the CHA mode excitation based on two steps, which consists of anapole and MQ modes. The near‐field distribution (left) and the far‐field scattering cross section (bottom right, SCS) of the SDR (top left, blue line) and the SSDR (bottom left, red line). A schematic of the SSDR is located on the top right. b) Illustration of THG from SSDRs at optical frequencies. The sample comprises a square array of SSDRs on fused silica substrate (SiO_2_). c) Scanning electronic microscopy image of an array of SSDRs from 45°‐tilted view. The disk radius *R*_D_, inner (*R*_in_) and outer radii (*R*_out_) of the ring, thickness (*T*), and period (*P*) are 230 nm, 390 nm, 490 nm, 100 nm, and 2 µm, respectively. An air slot with the length and the width of 260 nm and 30 nm is also introduced. The incidence is propagating along the *z*‐axis, the polarization is along the *y*‐axis (perpendicular to the slot).

Our THG investigations are performed in the silicon (Si) meta‐structure systems, duo to the good compatibility between Si and mature integrated semiconductor technologies as well as a large *χ* ^(3)^ of Si.^[^ ^42^, ^43^ ^]^ Besides the large *χ* ^(3)^, to obtain great *η*_THG_ also needs to create an ideal photonic mode which can support both large local electric field (EF) enhancements and low dissipation loss. So, we proposed a two‐step method to realize these two goals (**Figure** **1**a).

The first step is called the mode hybridization, in which the constructive hybridization of the normal anapole (NA) mode and MQ mode is realized by surrounding a Si disk (SD) with a nanoring, constructing the meta‐structure of Si disk‐ring (SDR) with the hybrid anapole (HA) mode. The NA mode is formed by the interference of electric dipole (ED) and toroidal dipole (TD) moments, so it naturally has good performances in localizing the near EF with low nonradiative loss. Therefore, after coupling with the MQ mode which exhibits a strong radiative response and narrow resonance linewidth, the HA mode can further reinforce the near‐field characteristics of the anapole mode, leading to obvious increments of EF enhancements as comparing with the NA mode. Next, in the second step of EF redistributions, a slot is introduced to the center of the Si disk to build the meta‐structure of slotted Si disk‐ring (SSDR), where the confined hybrid anapole (CHA) mode can be achieved (right part of Figure 1a). The CHA mode is characterized by the highly confinement near‐field energy inside the center slot, which leads to the redistribution of displacement current and the shortened optical path inside the SSDR, and finally causes the increasing and decreasing of EF enhancements and radiative loss, respectively. Therefore, comparing with the NA mode, the CHA can exhibit obviously larger EF enhancements and much smaller system loss, providing an outstanding platform for studying the THG process (Figure 1b).

Figure 1c shows the representative scanning electron microscopy (SEM) images of our samples, where the SSDRs are arranged in arrays with a period of *P* = 2 µm stratifying the conditions of disk radius *R*_D_ << *P*, ring outer radius *R*_out_< *P*, and incident light wavelength *λ* < *P*. Due to the setting of this large *P*, the near‐filed coupling between adjacent SSDRs can be negligible,^[^ ^34^ ^]^ which means it is reasonable to treat our sample as isolated SSDR.

### 2.2 The Formation Origin of the Confined Hybrid Anapole Mode

**Figure 2:**
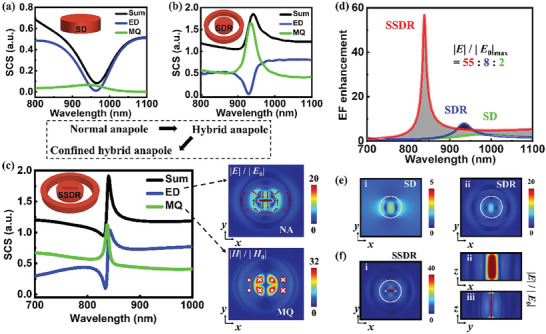
Optical responses of individual Si disk (SD), SDR and SSDR meta‐structures. a‐c) The contributions from multipole modes to the scattering spectra of the SD (a), SDR (b) and SSDR (c), respectively. They are electric dipole (ED, blue line), magnetic quadrupole (MQ, green line) and total contribution (Sum, black line). The extension diagrams show the electric (2c(i)) and magnetic (2c(ii)) field enhancements on the *z* = 0 plane at CHA mode. The arrows show the electromagnetic field vectors. d) The EF enhancement |*E*|/|*E*_0_| at the center of the nanodisk in three meta‐structures. e) The near‐field profiles on the *z* = 0 plane of the SD (i) and SDR (ii) at resonant wavelength. f) The near‐field profiles on the *z* = 0 (i), *y* = 0 (ii), and *x* = 0 (iii) plane of the SSDR, respectively. The parameters are the same as those in Figure 1.

Utilizing the Cartesian multipole decomposition based on the induced current, we conducted mode analysis on three systems of SD, SDR and SSDR, which respectively support NA, HA, and CHA modes (**Figure** **2**a–c). Herein, the ED (blue lines) contributions already include the common ED and TD terms,^[^ ^44^, ^45^ ^]^ showing the interaction of co‐excited ED and TD moments with same amplitude and opposite phase, i.e., the NA contributions. From the results of multipole decomposition, one can see a thorough mode evolution process and merits of the CHA mode.

First, the hybridization process from NA to HA mode can be observed as the resonant response evolves from valley to peak (see the black lines in Figure 2a,b). It stems from a significant increase in MQ contribution (green line), resulting in a change in the total far‐field characteristics. Such mode hybridization (i.e., NA + MQ) makes the HA mode show a large far‐field response with narrow linewidth and simultaneously strengthens the NA mode (i.e., the dip of ED spectrum become prominent, blue line), which leads to the decrease and increase of the radiative loss and EF enhancements of the HA mode, respectively. Second, EF redistribution is attributed to boundary conditions^[^ ^46^ ^]^ and mode leakage caused by the introduction of a slot and forms an evolution from HA to CHA mode. Compared to the HA mode (≈55 meV), the CHA mode exhibits sharper (≈20 meV) resonant peak because the linewidth of the MQ mode becomes narrower and the appearance of the Fano‐like spectrum of the ED mode (Figure 2c), which means lower loss (much smaller than previous results of isolated all‐dielectric meta‐structures^[^ ^47^, ^48^ ^]^). The pronounced Fano dip of ED spectrum implies that the light‐field confinement ability of the NA mode will be further improved, that is, greater EF enhancements.

In order to intuitively demonstrate the hybridization mechanism and near‐field characteristics of the CHA mode, the electric and magnetic field distributions on the *z* = 0 plane have been simulated (right part of Figure 2c). Two pairs of circular displacement currents occur simultaneously in the nanodisk and the nanoring, which means that two NA modes co‐exist. Additionally, two magnetic fields with opposite directions (± *z*‐axis) on each of the left and right sides of the profile. Such distribution is induced by the combination of NA and MQ modes, it agrees well with the multipole decomposition results. Thus, the maximum EF enhancement (|*E*|/|*E*_0_|_max_) ratio of three meta‐structures is 55: 8: 2, demonstrating that the CHA mode in SSDR can induce an astonishing EF enhancement which is comparable to plasmonic modes^[^ ^9^, ^49^, ^50^ ^]^(Figure 2d). In Figure 2e,f(i), we plot EF distributions on the *z* = 0 plane of three meta‐structures at resonant wavelengths, respectively. One can find that in the same spatial position, the SSDR has a strongest ability to confine the light‐field, resulting in maximal average EF enhancements over the whole SSDR meta‐structure (Figures 2f(ii‐iii)), and creating a larger nonlinear response region.

### 2.3 The THG Measurement Based on the Slotted Si Disk‐Ring Meta‐Structure

**Figure 3:**
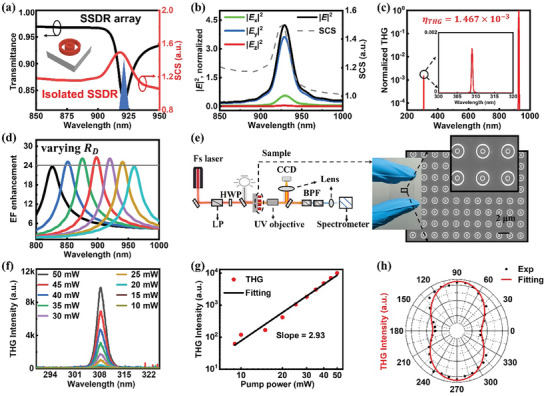
The simulated and experimental optical characterization of THG emission at the CHA mode. a) Simulated transmission (black) and SCS (red) spectra of the SSDR with the pump pulse spectrum denoted with the blue area. b) shows absolute squared EF (i.e., |*E*_x_|^2^, |*E*_y_|^2^, |*E*_z_|^2^, and |*E*|^2^) integrated over the volume of the SSDR. c) Simulated THG signals of the SSDR meta‐structure under pump pulse (near resonant wavelength). d) Curve of EF enhancement at the center of the slot as a function of radius (*R*_D_ = 210, 220, 230, 240, 250, 260, 270 nm) when other parameters are constant. e) Detection set up for collection of THG signals from SSDR array. The SEM image from top‐tilted view of the sample fragment. The scale bar is 2 µm. f) Measured spectra of THG under different pump powers at the CHA mode. g) Power dependence of the THG signal, the cure slope value of 2.93 indicates a third‐order nonlinear optical process. h) The measured THG intensity versus the polarization of pump laser.

Different from previous studies mainly focused on the THG emission in visible region, our study is aimed at expanding THG emission below 350 nm, shedding light on the design of integrated and on‐chip UV sources. So, in the beginning process of sample design, we should delicately make the SSDR with CHA mode resonating at ≈927 nm. Based on theoretical simulations, we can identify the structure parameters as that, the disk radius *R*_D_, inner (*R*_in_) and outer radii (*R*_out_) of the ring, and thickness (*T*) are 250 nm, 390 nm, 490 nm, and 100 nm, respectively. An air slot with the length and the width of 260 nm and 30 nm is also introduced. With those structure parameters, it is noticed that the transmission spectrum (black line) of SSDR array (*P* = 2 µm) can well correspond to the CHA mode in scattering spectrum (red line) of an isolated SSDR meta‐structure (**Figure** **3**a). By comparing the far‐field and near‐field between the periodic system and isolated SSDR, one can find that the influence of lattice resonance on the formation of the confined hybrid anapole mode is negligible. This fact indicates that the sample can be treated as isolated SSDR due to the negligible optical coupling through near‐field interactions (more discussions can be found in Figure S1, Supporting Information).

In order to predict the *η*_THG_ of the SSDR meta‐structure, we simulated the THG response of SSDR with silica substrate based on the experimental model. The distinct THG response is induced under pump pulse near the resonant wavelength. It can be well explained by integrating absolute squared value of time‐averaged field (${\left|E(\lambda )\right|}^{2}=\int \left({|E_{x}\left(r,\lambda \right)|}^{2}+{|E_{y}\left(r,\lambda \right)|}^{2}+{|E_{z}\left(r,\lambda \right)|}^{2}\right)dr\;$)^[^ ^34^ ^]^ over the volume of the SSDR (Figure 3b). The large |*E*|^2^ induces strong third‐order nonlinear polarization ($\tilde{P}\;\left(3\omega \right)=\chi_{Si}^{\left(3\right)}\;{\tilde{E}}^{3}\left(\omega \right)$),^[^ ^34^, ^51^ ^]^ which also implies large THG. We theoretically calculate the emission intensity of THG with a pump wavelength range of 800–1000 nm, and obtain Figure S2a (Supporting Information). It can be clearly found that the optimal THG emission efficiency is at the resonant wavelength. Based on these simulations, one can observe that there are no other resonant peaks near the CHA mode, and the enhanced THG signal mainly comes from the expected response (Figure 3c). The theoretical *η*_THG_ can be up to 1.47 × 10^−3^ due to ignoring realistic factors (e.g., the imperfection in the nanofabrication, collection efficiency, etc.), which is an impressive result showing the great potential of the SSDR meta‐structure for realizing strong THG signals. In addition, we also calculate the normalized THG signals of SD, SDR and SSDR at their respective resonance wavelengths, and the THG enhancement of SSDR meta‐structure is better than that of SDR and SD meta‐structures, as shown in Figure S2b (Supporting Information). Also, it is found that from the perspective of fabrication, the SSDR has the advantage of strong robustness against fabrication uncertainties. As shown in Figures 3d and Figure S3 (Supporting Information), the curves of different colors indicate the EF enhancements of CHA modes at different wavelength with varying *R*_D_ from 210 to 270 nm. Although the resonant wavelength of the CHA mode varies a span of over 130 nm, its EF enhancement at resonant and linewidth (*γ*) remain stable, i.e., |*E*|/|*E*_0_|_max_ > 24 (Figure 3d) and *γ*_1_ = *γ*_2_ = *γ*_i_. In Supporting Information, Figure S3 (Supporting Information) shows the wide tunability of the SSDR nanosystems in enhancing the THG signal. In addition, we conducted a detailed study on the effects of radiation loss of the photonic modes and absorption loss of material on THG, as shown in Figure S4 (Supporting Information).

Next, we experimentally measured the enhanced UV THG signal based on SSDR meta‐structures (details can be found in Optical Characterization). Figure 3e shows the measurement setup and the SEM image from top‐tilted view of the sample fragment. The diameter of the laser focusing spot is 7.48 µm, and such a spot contains ≈6–9 SSDRs. Under excitation with specific polarization (perpendicular to the slot), we examined the power dependence of the UV THG from the SSDR meta‐structures pumped at the wavelength of CHA mode. The measured THG spectra under different pump powers are displayed in Figure 3f, and the intensity of THG signal becomes more pronounced as the pump power gradually increases. Even the smaller pump power (i.e., 5 ≈ 9 mW) can also excite THG signals (the THG intensity plot versus low pump power is plotted in Figure S5, Supporting Information). Furthermore, the THG intensity as a function of the pump power is plotted in Figure 3g, exhibiting a line slope value of 2.93. The almost cubic dependence of the signal intensity on the pump power unequivocally verifies the nature of the third‐order nonlinear optical process.

Different polarizations affect the EF enhancements of SSDR, so the THG emission intensity changes with the polarization variation of incident light (Figure 3h). The THG emission reaches its weakest value when the incident polarization is parallel to the long side of the slot (*x*‐direction). It is consistent with our design depicted in Figure 1 that the CHA mode has the weakest response with the *x*‐polarized pump laser. However, the weakest intensity of the THG remains above 1/3 of the maximum value which is obtained with *y*‐polarized pump laser (i.e., the incident polarization is vertical to the long side of the slot), showing the EF enhancement of our CHA mode possesses a relatively good property of polarization independence of incident light.

### 2.4 The Measurement of THG Conversion Efficiency

**Figure 4:**
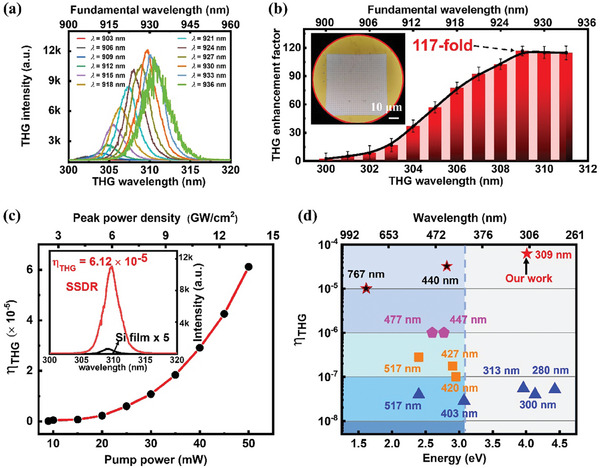
Employment of the CHA mode for THG enhancement. a) Measured spectra of THG when scanning the pump wavelength across center wavelength of the CHA mode under the pump power of 50 mW. b) Measured THG enhancement factor (THG_SSDR_ / THG_film_) of the SSDR meta‐structure with different excitation wavelengths. The inset shows a microscopic image of structural and non‐structural areas of the sample. c) The achieved conversion efficiency as a function of the pump power. The inset shows THG spectra of the SSDR meta‐structure and Si film with same thickness measured at excitation wavelength of 927 nm. d) The experimental *η*_THG_ of our SSDR meta‐structure and comparisons with previous all‐dielectric Si‐based meta‐systems.^[^ ^28^, ^29^, ^30^, ^33^, ^34^, ^36^, ^38^, ^51^, ^55^, ^56^, ^57^, ^58^ ^]^ The triangles, squares, pentagons, and stars correspond to works with *η*_THG_ at the order of magnitude of 10^−8^, 10^−7^, 10^−6^, and 10^−5^, respectively.

To experimentally find the optimal excitation wavelength for THG of our SSDR meta‐structure, as well as investigating the positive effect of the CHA mode on inducing THG signal, we study the dependence of the THG spectra with the excitation wavelength (**Figure** **4**a). One can see that the strongest THG signal appears when the pump wavelength coincides with the resonant CHA mode (i.e., ≈927 nm), and the THG signal is weakened in the narrow‐band (corresponding to narrow linewidth of the CHA) of the offset resonant wavelength. The dependence relationship between THG intensity and excitation wavelength can be more clearly illustrated by Figure 4b, where we measured and calculated the THG enhancement factors with fundamental wavelength ranging from 900 to 933 nm. The enhancement factor is calculated by the ratio of THG signal of the SSDR metasurface to pure Si nanofilm with the same thickness (≈100 nm) and being deposited in the same batch (i.e., THG_SSDR_/THG_film_). One can observe that as the fundamental wavelength deviates from resonant wavelength of the CHA mode, the enhancement factor decreases. Even with a deviation of only 20 nm, the enhancement factor decreases from 117 times to below ten times. The inset shows a microscopic image of structural and non‐structural areas of the sample, where the gray area is the array of SSDR meta‐structures.

The *η*_THG_ is measured by the equation *η*_THG_ = *P*_3ω_/*P*_ω_, where *P*_3ω_ and *P*_ω_ are the THG power and the pump power, respectively (calculation details are given in Supporting Information Note 1). Due to the cubic law dependence between *η*_THG_ and *P*_ω_ (Figure S5, Supporting Information), the *η*_THG_ is expected to grow with the increasing of excitation power.^[^ ^28^, ^52^ ^]^ So, we measured the dependence relationship of *η*_THG_ and the excitation power, with setting incident wavelength of 927 nm (Figure 4c). The incident peak irradiance varied from 2.4 to 13.3 GW cm^−2^, which is a suitable and widely‐applied power range for studying THG in nanosystem. According to previous studies,^[^ ^28^, ^38^ ^]^ the power density within this power range of excitation laser can ensure that a strong THG signal is pumped out without exceeding the two‐photon absorption threshold of silicon, so such power range is widely‐used. Based on our experimental results, when the incident peak irradiance is 13.3 GW cm^−2^ (50 mW), the maximal measured efficiency of near‐infrared‐to‐UV conversion is extracted as high as 6.12 × 10^−5^ at the emission wavelength of 309 nm.

The obtained in our SSDR meta‐structure system is an impressive result (Figure 4d; Table S1, Supporting Information). Firstly, it is noticed that the thickness of our SSDR is only 100 nm, which is the thinnest structure in the all‐dielectric system for THG investigations. Such thin thickness can facilitate the integration of large‐scale and high‐density devices. Secondly, different from the majority of THG studies in all‐dielectric systems which focus on the emission in visible region, our results demonstrate that UV emission below 350 nm can be easily realized. Thirdly, to the best of our knowledge, for the all‐dielectric nanosystems the *η*_THG_ for UV emission below 350 nm is at the order of ≈5.5 × 10^−8^ (the excitation power is ≈40 mW).^[^ ^33^, ^34^ ^]^ Comparing with these results, our results have notably improved the *η*_THG_, greatly promoting the application of nanophotonic nonlinear devices in UV region.

Beyond the high *η*_THG_, a more important finding is that we propose the idea of a two‐step strategy for obtaining ideal photonic mode with both high EF enhancements and low loss for investigating light–matter interactions. The two steps are named as the mode hybridization and EF redistribution, respectively. In these two steps, the physical essence of mode hybridization is the interference between different photonic modes, which can suppress the leakage channels, thereby reducing the radiation loss of photonic modes. On the other hand, the EF redistribution means that by introducing a discontinuity in the internal uniform refractive index (RI) environment of a photonic cavity, the charge can be concentrated toward the interface of the RI jump, leading to great enhancements of localized EFs. The above two steps can be utilized independently or in combination. Hence, with the employment of this two‐step strategy, our obtained CHA mode can have both strong EF enhancements (≈50‐folds) and small system loss (≈20 meV) without substrate. The EF enhancement is comparable with plasmonic nanocavities,^[^ ^53^, ^54^ ^]^ and the loss is much lower than previous isolated all‐dielectric nanocavities.^[^ ^47^, ^48^ ^]^ Therefore, when the CHA mode is applied for THG investigation, we can obtain the largest experimental conversion efficiency as high as 6.12 × 10^−5^ at the UV band of 309 nm in the all‐dielectric nanosystems. In fact, besides the investigation in nonlinear optics, our idea of a two‐step strategy can be applied to construct photonic modes for other studies of light–matter interactions, such as the cavity quantum electrodynamics (c‐QED), emission enhancement, nanolaser, etc.

## 3 Conclusion

In conclusion, we have proposed a two‐step approach to construct the photonic mode which can simultaneously have strong EF enhancements and small system loss. The two steps are the mode hyperdilation and EF redistribution, based on which we have creatively built a novel CHA mode that is originated from the constructive coherence of NA and MQ modes in the SSDR meta‐structure system. The CHA mode can theoretically bring about EF enhancements over 50‐folds which is comparable to plasmonic modes, as well as a small system loss of ≈20 meV which is much smaller than previous records of isolated all‐dielectric meta‐structures. When the SSDR meta‐structure is applied for THG investigations, remarkable advances can be obtained due to the existence of CHA mode. For one thing, a high *η*_THG_ of 6.12 × 10^−5^ has been achieved at the emission wavelength of 309 nm, due to the resonance between CHA mode and excitation light. Comparing with the experimental *η*_THG_ of emission wavelengths shorter than 350 nm in the all‐dielectric nanosystems, our *η*_THG_ is a new record, as far as we are concerned. Furthermore, our SSDR meta‐structure is only 100 nm in thickness, which is two–five times thinner than most previous all‐dielectric nanosystems applied in THG studies. Such thin thickness can greatly benefit for the integration and miniaturization of nonlinear nanodevices. Our findings not only demonstrate a thin meta‐structure with highly efficient THG emission in UV region, but also provide the idea to establish the ideal photonic platform for studying light–matter interactions, which can pave the way for designing and fabricating advanced photonic and optoelectronic nanodevices.

## 4 Experimental Section

### Sample Fabrication

To achieve the desired enhancement of THG by confined hybrid anapole (CHA) mode in amorphous silicon (a‐Si) nanostructure, the design depicted in Figure 1c is proposed. The sample fabrication process consists of two parts: The deposition of a‐Si and nanostructure fabrication. 1) Deposit 100 nm‐thick a‐Si layer on a fused silica substrate using an inductively coupled plasma chemical vapor deposition (ICPCVD) process with 10 sccm of SiH_4_ and 20 sccm of 10%B_2_H_6_/Ar mix at 300 °C, 8 mTorr chamber pressure and 1 W reflected power. 2) Spin‐coating 140 nm Hydrogen Silsesquioxane (HSQ) on the a‐Si film with 4000 rpm for 1 min and baked at 90 °C for 3 min. 3) A 40 nm layer of conductive aluminum was steamed onto the sample surface. 4) Electron beam lithography (EBL) was used to define a mask for the subsequent pattern transfer. Fabrication nano‐structure by EBL at 100 keV with an area dose of 1700 µC cm^−2^. After EBL, the sample was soaked in a water bath at 55 °C with 5% phosphoric acid solution for ≈8 min until the Al layer on the HSQ surface was removed. Then, soak in tetramethylammonium hydroxide solution for 2 min, soak in deionized water for 1 min, and blow‐dry with nitrogen. 5) Etch by inductively coupled plasma (ICP) and get the a‐Si structure. The hydrogen bromide (HBr) gas with a flow rate of 20.1 sccm was ignited using RF forward power of 57 W and ICP power of 503 W in a chamber pressure of 3.9 mTorr at 21 °C. The rate of etching a‐Si was ≈3.34 nm s^−1^, and finally the SSDR metasurface with a thickness of 100 nm was obtained. The instrument model and brand for ICP etching mentioned above was PlasmaPro System 100ICP180, Wavetest; for EBL was EBPG5000+, Raith; and for ICPCVD was PlasmaPro System100 ICP180‐CVD, Oxford.

### Optical Characterization

As shown in Figure N1, the femtosecond laser beam was generated from an optical parametric oscillator (MaiTai HP OPIUM AUTO OPO) with a pulse width of 100 fs and a repetition rate of 80 MHZ in Figure 3e. The THG signal generated by the sample was sequentially focused into the high sensitivity and resolution spectrometer (JOBIN YVON Triax550) through the UV objective (NA = 0.13) and two bandpass filters (BPF, OD > 4.0), in order to obtain a relatively pure THG signal.

### Numerical Simulations

The numerical simulation was calculated by finite‐difference time‐domain software (FDTD solutions, Lumerical Inc.). For the isolated Si‐based SSDR meta‐structure, the excitation source was total‐field scattered‐field (TFSF) wave. To simulate an individual structure placed in an infinite space, perfectly matched layer (PML) boundary conditions were used. A power monitor box is setted consisting of six 2D monitors in scattered light region. By adding the 6 transmissions and multiplied by the source area one could get the scattering cross section. For the SSDR array, the excitation source was plane wave and periodic boundary conditions were used. A 2D DFTmonitor capable of collecting Pointers vectors and power was used to obtain transmission signals. In all simulations, the mesh sizes around the air slot and the SSDR structure were 1*1*1 nm^3^ and 4*4*4 nm^3^, respectively. The surrounding index for simulations is *n* = 1 and the dielectric constant of the Si is taken from “Silicon‐Palik” in the FDTD Materials Database. The detailed methods of multipole decompositions are shown in Note S2 (Supporting Information). Considering the residual photoresist during the electron beam lithography (EBL) processing, a silicon oxide (refractive index *n* = 1.46) layer of 40–70 nm was added at top of the SSDR during numerical simulation to better match the actual situation. In fact, the influence of lithography residues on the EF distribution of the SSDR could be negligible. In simulation, the nonlinear susceptibility tensor *χ*_Si_^(3)^ was considered as a constant scalar value of *χ*_Si_^(3)^ = 2.45 × 10^−19^m^2^ V^−2^ which was similar to previous studies.^[^ ^30^, ^43^, ^59^ ^]^

## Conflict of Interest

The authors declare no conflict of interest.

## Supporting information

Supporting Information

## Data Availability

The data that support the findings of this study are available in the supplementary material of this article.
