# Supplementary material for: Highly Efficient Ultraviolet Third‐Harmonic Generation in an Isolated Thin Si Meta‐Structure
Source: Adv Sci (Weinh). 2024 Jul 8;11(34):2404094. doi: 10.1002/advs.202404094 (PMC11425875; doi:10.1002/advs.202404094)
Supplement: Supplementary file 1 — Supporting Information [file ADVS-11-2404094-s001.docx]

Supporting Information for

**Highly Efficient Ultraviolet Third-Harmonic Generation in an Isolated Thin Si Meta-Structure**

Yanhui Deng^1^, Zhonghong Shi^1^, Yaqin Zheng^1^, Houjiao Zhang^1^, Haoyang Li^1^, Siyang Li^1^, Zhang-Kai Zhou^1,*^

^1^State Key Laboratory of Optoelectronic Materials and Technologies, School of Physics, Sun Yat-sen University, Guangzhou 510275, China

*Corresponding Author: [zhouzhk@mail.sysu.edu.cn](mailto:zhouzhk@mail.sysu.edu.cn)

**Contents**

Figure S1. Near-field comparison between isolated SSDR and SSDR array.

Figure S2. Simulated THG signal enhancement.

Figure S3. The great robustness of the SSDR meta-structure.

Figure S4. Loss Analysis.

Figure S5. The power dependence of the THG signal.

Note 1. The calculation of the THG conversion efficiency.

Note 2. Cartesian multipole decomposition for SD, SDR and SSDR.

Table S1. THG conversion efficiency of the all-dielectric structures.

**Figure S1. Near-field comparison between isolated SSDR and SSDR array.**

**
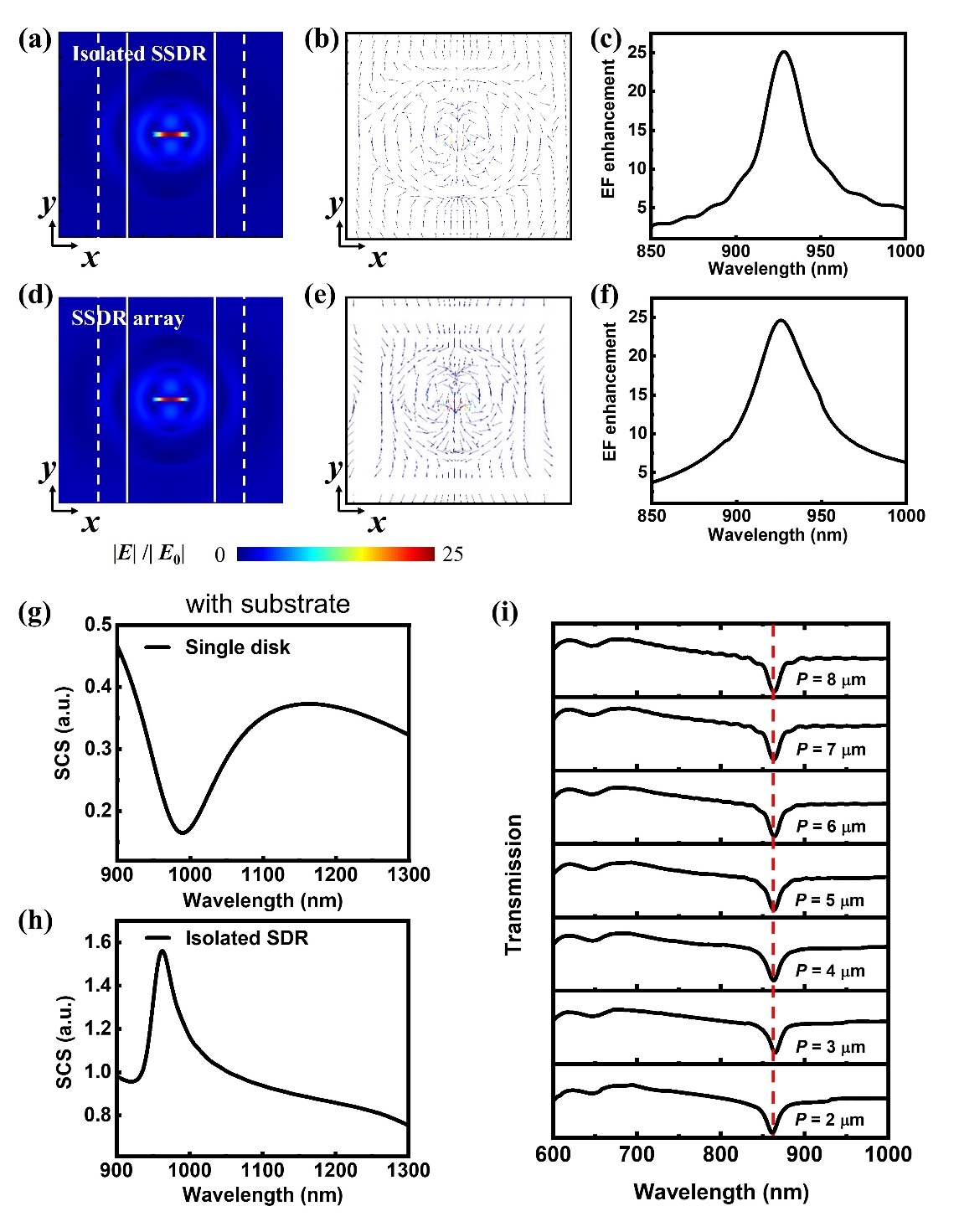
**

**Figure S1** Near-field comparison between isolated SSDR and SSDR array**.** (a, d) The near-field profiles on the *z* = 0 plane of the (a) isolated SSDR meta-structure and (d) SSDR array at the resonant wavelength. (b, e) Charge distribution on the *z* = 0 plane of the (b) isolated SSDR meta-structure and (e) SSDR array excited by normal incidence plane wave with polarization along the *y* axis. (c, f) The EF enhancement in the center of the slot in the (c) isolated SSDR meta-structure and (f) SSDR array, respectively. All parameters are the same as those in Figure 3 of the main text. (g) The scattering efficiency of a single disk. (h) The scattering efficiency of an isolated SDR. The dimensional parameters of the meta-structures of SD and SDR on SiO_2_ (*n* = 1.46) substrates are the same as those in Figure 2, except for the addition of a substrate. (i) Transmission spectra of SSDR arrays with different periods *P*. For convenience, the substrate was not added

To further illustrate that the optical coupling between the SSDR meta-structures in the array due to near-field interactions can be neglected with such a period (*P* = 2 *μm*), the near-field distributions and electric field (EF) enhancements of isolated SSDR meta-structure and SSDR array at their respective resonances were compared. The most important point has been presented in Figure 3a in the main text, namely that the resonant wavelengths of isolated SSDR and SSDR array are the same.

In Figure S1, three obvious optical features can fully illustrate this equivalence. Firstly, for the representative plane (*z* = 0), the distribution and intensity of the near field is the same both inside and outside the SSDR (Figures S1a and S1d). Secondly, there are two pairs of circular displacement currents in each of the two EF vector profiles, which means that the resonance behavior is the designed confined hybrid anapole (CHA) mode (Figures S1b and S1e). Thirdly, the trend and value of EF enhancements in the slot center of isolated SSDR and SSDR array are same, which means that there is no optical response enhanced by near-field coupling in SSDR array, or the interaction between SSDR meta-structures can be ignored (Figures S1c and S1f). Therefore, we can treat our sample as isolated SSDR.

Based on these results, one can find that the influence of lattice resonance on the formation of the CHA mode is negligible. Firstly, for a period of *P* = 2 *μm*, the resonance wavelengths, electric field enhancements, and charge distributions of an isolated SSDR and SSDR array are almost identical. Secondly, for lattice resonance, the resonance wavelength should shift with the variation of the period. But, Figure S1i shows that the resonance position of the SSDR array remains unchanged at different periods. Therefore, when the period *P* = 2 *μm*, the effect of lattice resonance can be ignored, and we can equivalent the SSDR array to an isolated SSDR meta-structure. In addition, it was mentioned in the literature^[1]^ that the optical coupling through near-field interaction can be ignored for an array with diameter *d* << *P* and resonant wavelength *λ* < *P*. For the SSDR system whose resonant wavelength is dominated by the nanodisk (Figure S1g and h), the approximate conditions described in the literature are satisfied (i.e., *R*_D_ << *P* and *λ* < *P*).

**Figure S2. Simulated THG signal enhancement.**

In this paper, the nonlinear emission of THG is simulated in two steps using the time-domain finite-difference (FDTD solutions, Lumerical Inc.) method. In the first step, we simulate the linear optical transmission and 3D field-constrained enhancement of the proposed meta-structures near the fundamental frequency *ω*. In the second step, we calculated the third-order electric nonlinear polarization $\tilde{P}\left( 3\omega\right)=\varepsilon_{0}\chi_{Si}^{\left( 3 \right)}\tilde{E}^{3}(\omega)$ at the THG frequency.^[2,3]^ In simulation, $\varepsilon_{0}$ = 8.85 🞨 10^-12^ F/m is the air permittivity, the incident electric field amplitude of 1🞨10^9^ V/m, the pulse duration of 100 fs, the nonlinear susceptibility tensor *χ*_Si_^(3)^ is considered as a constant scalar value of *χ*_Si_^(3)^ = 2.45 🞨 10^-19^ m^2^/V^2^.^[4]^ The normalized THG emission spectrum of the a-Si meta-structures are shown in Figure S2.


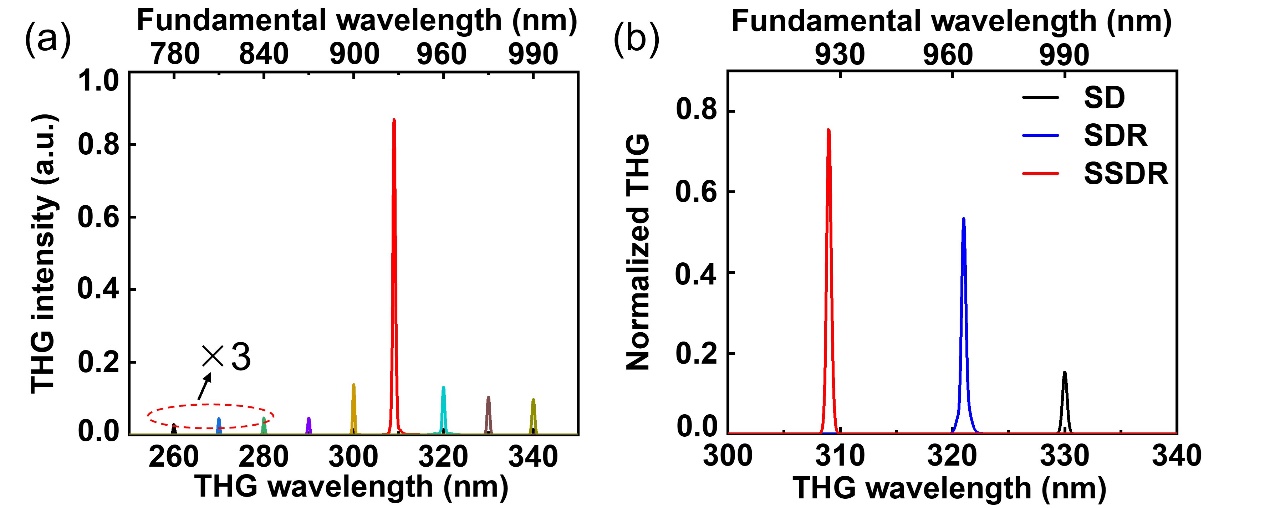


**Figure S2** (a) THG signal enhancement of isolated SSDR under the pump wavelength range of 800-1000 nm. (b) THG signal enhancement of SD, SDR, and SSDR meta-structures under resonant wavelength excitation, respectively. Silicon nanodisk with anapole mode is a promising candidate for achieving THG enhancement,^[5]^ which possesses the ability to highly localize the optical field within the dielectric cavity. By combining the advantages of anapole and magnetic quadrupole modes (i.e., improving the electric field and reducing the radiation loss of the cavity mode), the SSDR meta-structure enables the THG signal of silicon-based systems to be further improved by a factor of 5 as comparing with the SD with the anapole mode.

**Figure S3. The great robustness of the SSDR meta-structure.**

**
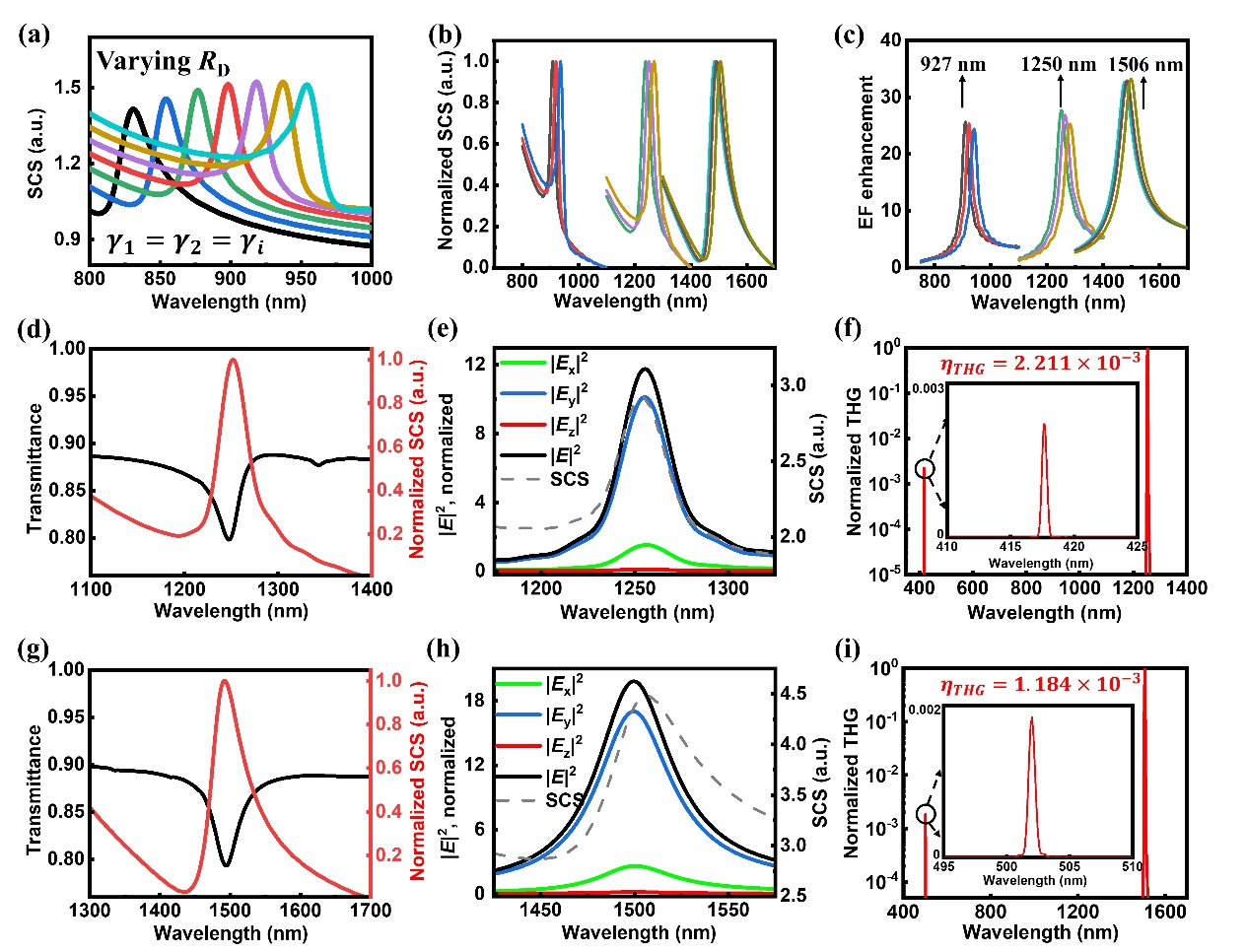
**

**Figure S3** The great robustness of the SSDR meta-structure. (a) Curve of EF enhancement at the center of the slot as a function of radius (*R*_D_ = 210, 220, 230, 240, 250, 260, 270 nm) when other parameters are consistent with those in Figure 3a of the main text. (b) Normalized SCS spectra for SSDR Of different sizes with resonant peaks around 927,1250 and 1506 nm. (c) The EF enhancement at the center of the slot varies with wavelength. (d, g) Simulated scattering (red) transmittance (black) spectra of the SSDRs with different sizes. (e, h) show absolute squared EF (i.e., |*E*_x_|^2^, |*E*_y_|^2^, |*E*_z_|^2^, and |*E*|^2^) integrated over the volume of two SSDRs, respectively. (f, i) Simulated THG signals of two SSDR meta-structures under pump pulse (near the resonant wavelengths).

In Figure S3, we show the robustness of the SSDR meta-structure in enhancing the THG signal. As shown in Figure S3a, the curves of different colors indicate the tuning of the radius *R*_D_ (from 210 to 270 nm). Although the resonant wavelength of the CHA mode has a span of over 130 nm, its linewidth is almost constant, i.e., *γ*_1_ = *γ*_2_ = *γ*_i_. To broaden the working wavelength of the THG, we adjust geometric parameters and tune wavelengths of the CHA mode from 927 to 1250 to 1506 nm (i.e., proportional scaling). One can find that SSDRs with CHA mode always have high EF enhancement (> 24) and almost constant system loss (Figure S3b and S3c). The curves of different colors indicate the fine tuning of the radius *R*_D_, that is, the dimensional error of the processing. Meanwhile, there are no other resonant peaks near the CHA mode, and the enhanced THG signal mainly comes from the expected response.

To achieve wide modulation of the fundamental wavelength. In Figures S3d and S3g, numerical simulations for linearly polarized light at normal incidence are performed with finite difference time-domain (FDTD) method. One can find that the spectrum redshifts with increasing size as expected and that the overall shapes and trends of the transmittance spectra (black lines) are in reasonable agreement with the scattering spectra (red lines). The SSDR array with a large period can be treated as an isolated SSDR due to the negligible optical coupling through near-field interactions.

As expected, our SSDR also theoretically enables the enhancement of THG signals of IR-to-visible. At resonant wavelengths of 1250 and 1506 nm, there is also the largest |*E*|^2^ (Figure S3e and S3h), and the THG signals generated near 417 and 502 nm, respectively. In Figure S3f, 3c and S3i, one can find that the conversion efficiency of THG with different emission wavelengths is ordered as THG_417_ > THG_309_ > THG_502_. With the pump wavelength redshift, on the one hand, the radiation loss and electric field enhancement of the resonant photonic mode gradually increase. On the other hand, the absorption loss of the material as a function of the wavelength (Figure S4b). Specifically, compared with the THG_309_, the THG_417_ corresponds to a larger electric field enhancement at fundamental-frequency (Figure S3c), lower pump absorption loss, as well as emission wavelength loss and radiation losses (Figure S3b) close to THG_309_. Therefore, the conversion efficiency of THG_309_ is lower than that of THG_417_. THG_309_ is slightly higher than THG_502_ mainly due to the fact that THG_309_ has significantly smaller radiation loss of the photonic mode. In conclusion, the photonic mode CHA induced in SSDR meta-structure is robust in enhancing THG signal. The theoretical conversion efficiency *η*_THG_ can reach 10^-3^ when the incident electric field amplitude of 1🞨10^9^ V/m, When the pump power density is reduced, i.e., the incident electric field amplitude is 1🞨10^7^ V/m,^[3]^ the THG conversion efficiency is 10^-5^ (Figure S4d).

**Figure S4.** **Loss Analysis.**

**Radiation loss of photonic mode:** For the same nonlinear material, the radiation loss of photonic mode, i.e., the linewidth, has an important effect on the conversion efficiency. The radiation loss of common symmetric line shapes can be obtained by fitting a Lorentzian model. Radiation loss for asymmetric line shapes is generally fitted using the two-oscillator Fano-like model.^[6]^ After fitting based on the two-oscillator Fano-like model, the loss linewidths of SD, SDR and SSDR are 114 meV (91 nm), 55 meV (41 nm), and 26 meV (18 nm), respectively, as shown in Figure S4a. One can find that the normalized THG intensity gradually increases as the radiation loss of the photonic mode gradually decreases while keeping the same amplitude of the incident electric field, which also implies that the conversion efficiency increases with the decrease of the radiation loss (Figure S2b).

**Two-oscillator Fano-like model:** The scattering spectrum of the confined hybrid anapole (CHA) mode can be fitted by employing a Fano-like line shape, i.e. $E\left( \omega\right)={|e(\omega)|}^{2}$.^[6]^

$e\left( \omega\right)=a_{r}+\sum_{j=1,2} \frac{b_{j}\gamma_{j}e^{i\phi_{j}}}{\omega-\omega_{j}+i\gamma_{j}}$ (1)

where $a_{r}$, $b_{j}$, $\gamma_{j}$, $\phi_{j}$ and $\omega_{j}$ are constant, amplitude, radiative damping, phase, and resonant energy of the *j* different oscillators in the resonant state. A two-oscillator (*j* = 1, 2) model is employed to fit the scattering or extinction spectrum. Here, the dielectric cavity is described as two harmonic oscillators whose resonant frequencies are far apart. One of the oscillators is at the same frequency as the resonant mode and dominates the radiation loss of the mode, while the other affects the asymmetric line shape of the resonant mode.

**
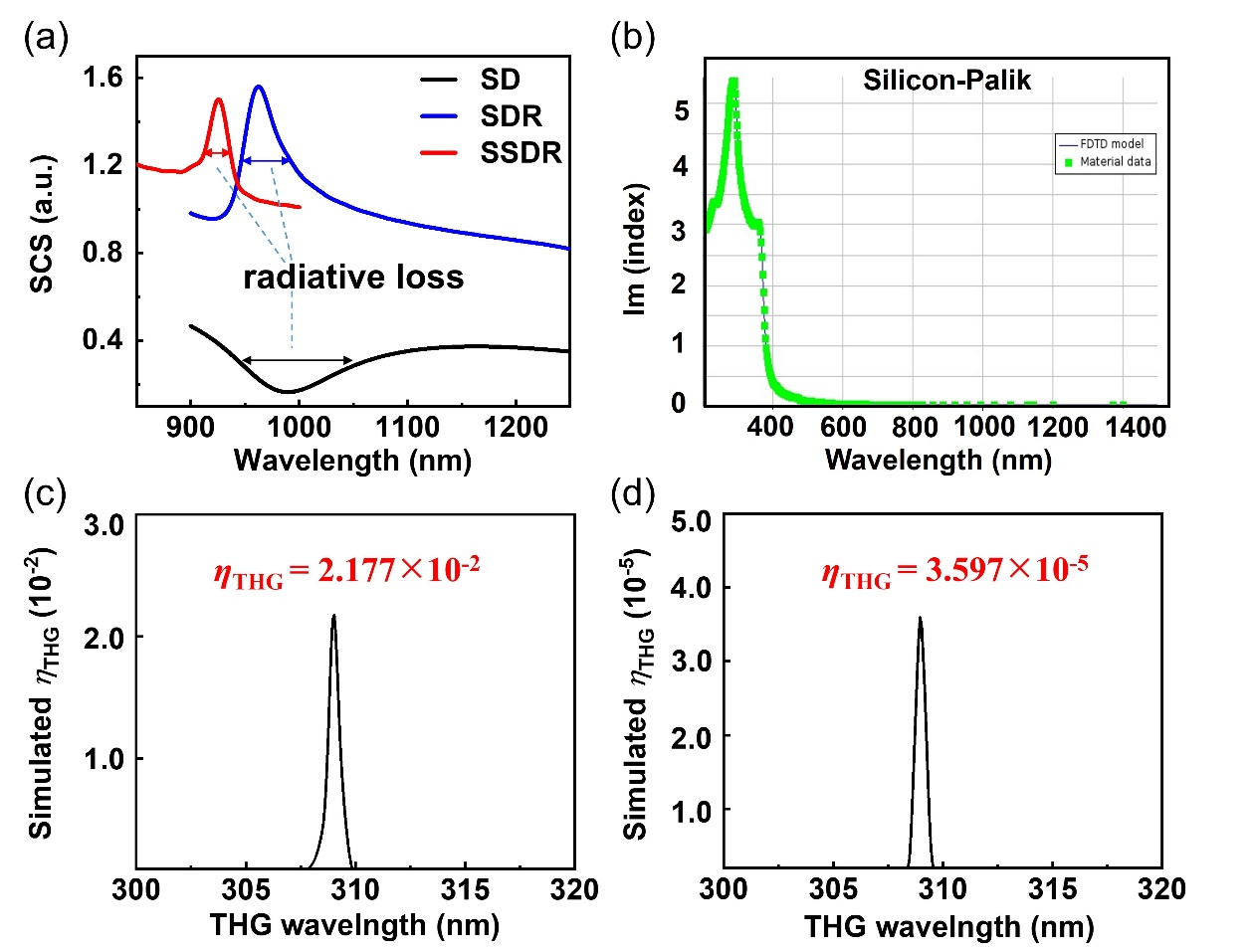
**

**Figure S4** (a) The scattering cross section of the SD (black line), the SDR (blue line) and the SSDR (red line). (b) Absorption loss of silicon from 200 nm to 1500 nm band.^[7]^ (c) THG conversion efficiency of the lossless SSDR meta-structure, when the incident electric field amplitude is 1🞨10^9^ V/m. (d) THG conversion efficiency of the SSDR meta-structure, when the incident electric field amplitude is 1🞨10^7^ V/m. These meta-structures are located on SiO_2_ (*n* = 1.46) substrate.

**Absorption loss of material:** In the simulation, the third-order susceptibility is set based on "Silicon-Palik" in the material library. The refractive index of the material is complex number and as a function of wavelength (Figure S4b), where the imaginary part of the refractive index characterizes the absorption loss of the material. As shown in Figure S3b, the linewidths of the modes at resonance wavelengths of 927 nm and 1250 nm do not differ much, assuming that the radiation loss is close. With the redshift of the pump wavelength, the absorption loss of silicon gradually decreases, and the THG conversion efficiency corresponding to 1250 nm is significantly higher, as in Figure S3f and Figure 3c. In addition, when the refractive index of silicon is a constant value of *n* = 3.6, which indicates a lossless material, the conversion efficiency can be as high as 10^-2^ under same power density (Figure S4c).

**Figure S5. The power dependence of the THG signal.**

**
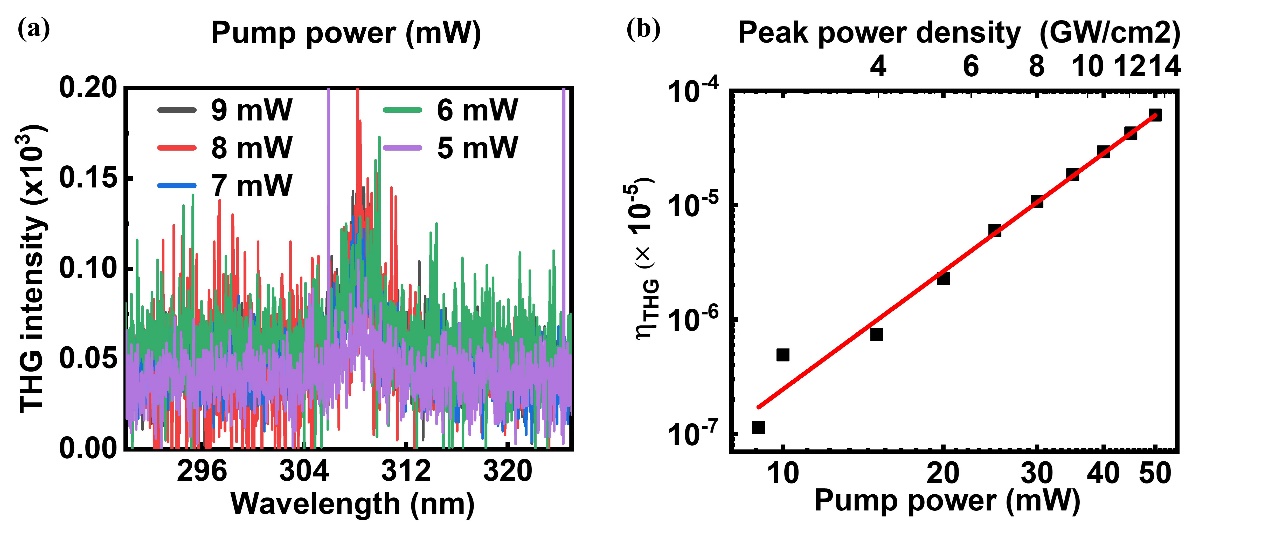
**

**Figure S5** The power dependence of the THG signal. (a) shows THG signal under low pump power (from 5 to 9 mW). (b) Power dependence of the conversion efficiency *η*_THG_ in SSDR meta-structure.

The small pump power (i.e., 5 ~ 9 mW) can also induce THG signals, as shown in Figure S5a. The result implies that our designed SSDR meta-structure with a CHA mode also possess the property of inducing THG signals with low pumping power, which will provide good opportunities for hybrid plasmonic-dielectric meta-structures to achieve nonlinear responses. In Figure S5b, a slope of about 3 indicates the cubic law dependence between conversion efficiency *η*_THG_ and pump power.

**Note 1. The calculation of the THG conversion efficiency.**


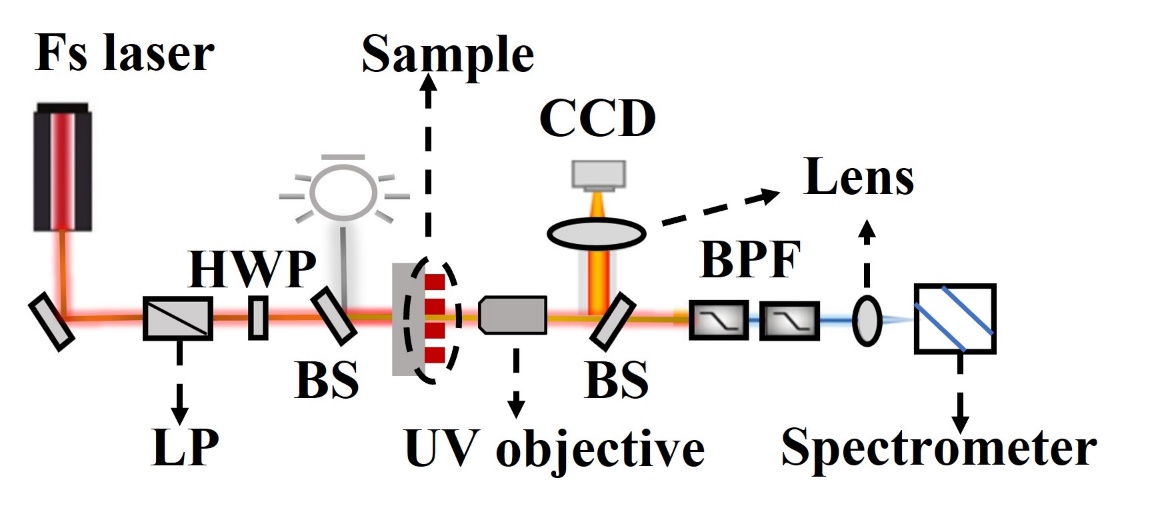


**Figure N1**. Schematic of experimental setup for the measurement of THG.

**The conversion efficiency of THG.** Firstly, we recorded the pump power *P*_ω_ at the front of the sample, and obtained the integral counts *I*_3ω_ of THG from the SSDRs. Under the same measurement condition, the integral counts *I*_ω_ of the mixed pump signal in THG is also obtained. At the same time, we recorded the total signal power *P*^(0)^ before entering the spectrometer with a Vega power meter with a highly sensitive picowatt probe. The fs laser we used is Mai Tai HP with a laser pulse length *τ* of 100 fs and the laser repetition rate *υ* of 80 MHz. Secondly, in order to obtain a relatively pure THG signal, the THG signal generated by the sample is sequentially passed through the UV objective (NEWPORT U-13X-LC, NA = 0.13), a beam splitter (BS, THORLABS BSW20R, transmission efficiency 50 %), two UV bandpass filters (EDMUND BPF #12-094, OD > 4.0), as well as a lens and finally focused into the high sensitivity and resolution spectrometer (JOBIN YVON Triax550). Thirdly, to calculate the power *P*_3ω_ of the THG signal, where the ratio of the quantum efficiency of the spectrometer (including the grating and the CCD) to the THG and the pump signal is *T*_s_. Then, the power entering the spectrometer is $P_{3\omega}^{(0)}=\frac{P^{(0)}I_{3\omega}}{I_{3\omega}+I_{\omega}T_{s}}$. In addition, the transmittance of UV, BS, BPF and lens to THG signal is *T*_U_, *T*_B_, *T*_BP_ and *T*_L_, respectively. So that the THG power from our sample can be obtained as $P_{3\omega}=\frac{P^{(0)}}{T_{U}T_{B}T_{BP}^{2}T_{L}}$. Finally, the nonlinear conversion efficiency can be calculated as *η*_THG_ = *P*_3ω_ / *P*_ω_.

**Note 2. Cartesian multipole decomposition for SD, SDR and SSDR.**

**Multipole decomposition method.** We calculate the contribution of electric dipole (ED), magnetic dipole (MD), electric quadrupole (EQ) and magnetic quadrupole (MQ) moments to the scattering cross section (SCS) of meta-structures based on the multipole decomposition method. Multipole moments can be expressed by adopting the induced currents $\hat{J}_{\omega}\left( \hat{r} \right)$ in the long-wavelength approximation:^[8-10]^

ED moment:

$p_{\alpha}=-\frac{\text{1}}{i\omega}\left\{ \int d^{\text{3}}\hat{r}J_{\alpha}^{\omega}j_{0}(kr)+\frac{k^{\text{2}}}{\text{2}}\int d^{\text{3}}\hat{r}[\text{3}\left( \hat{r}\cdot\hat{J}_{\omega} \right)r_{\alpha}-r^{\text{2}}J_{\alpha}^{\omega}]\frac{j_{\text{2}}(kr)}{({kr)}^{\text{2}}} \right\}$, (2)

MD moment:

$m_{\alpha}=\frac{\text{3}}{\text{2}}\int d^{\text{3}}\hat{r}{(\hat{r}\times\hat{J}_{\omega})}_{\alpha}\frac{j_{\text{1}}(kr)}{kr}$, (3)

EQ moment:

$Q_{\alpha\beta}^{e}=-\frac{\text{3}}{i\omega}\left\{ \int d^{\text{3}}\hat{r}{[\text{3}(r_{\beta}J}_{\alpha}^{\omega}+r_{\alpha}J_{\beta}^{\omega}-\text{2}\left( \hat{r}\cdot\hat{J}_{\omega} \right)\delta_{\alpha\beta}]\frac{j_{\text{1}}(kr)}{kr}+\text{2}k^{\text{2}}\int d^{\text{3}}\hat{r}[\text{5}r_{\alpha}r_{\beta}\left( \hat{r}\cdot\hat{J}_{\omega} \right)-\left( r_{\alpha}J_{\beta}+r_{\beta}J_{\alpha} \right)r^{\text{2}}+r^{\text{2}}\left( \hat{r}\cdot\hat{J}_{\omega} \right)\delta_{\alpha\beta}]\frac{j_{\text{3}}(kr)}{{(kr)}^{\text{3}}} \right\}$, (4)

MQ moment:

$Q_{\alpha\beta}^{m}=\text{15}\int d^{\text{3}}\hat{r}\left\{ r_{\alpha}{(\hat{r}\times\hat{J}_{\omega})}_{\beta}+r_{\beta}{(\hat{r}\times\hat{J}_{\omega})}_{\alpha} \right\}\frac{j_{\text{2}}(kr)}{{(kr)}^{\text{2}}}$, (5)

where $\alpha，\beta=x，y，z$, while *ω*, *k*, *c*, $\hat{r}$ are frequency, wavenumber, speed of light, and location, respectively. The induced electric current can be obtained by $\hat{J}_{\omega}\left( \hat{r} \right)=i\omega\varepsilon_{0}(\varepsilon_{r}-\text{1})E_{\omega}\left( \hat{r} \right)$, where $E_{\omega}\left( \hat{r} \right)$ is the electric field distribution, $\varepsilon_{0}$ is vacuum permittivity, and $\varepsilon_{r}$ is the relative permittivity. We utilize the simulation results of FDTD to obtain the electric field (EF) distributions $E_{\omega}\left( \hat{r} \right)/E_{inc}$, where $E_{inc}$ is the incident EF amplitude. The $j_{\text{1}}\left( kr \right),$ $j_{\text{2}}\left( kr \right)$ and $j_{\text{3}}\left( kr \right)$ are the modified spherical Bessel functions of first, second and third kinds,^[11]^ respectively. The SCS generated by multipole moments can be written as:

$$C_{sca}^{total}=C_{sca}^{p}+C_{sca}^{m}+C_{sca}^{Q^{e}}+C_{sca}^{Q^{m}}+\cdots$$

$=\frac{k^{\text{4}}}{\text{6}\pi{\varepsilon_{0}}^{\text{2}}{{|E}_{inc}|}^{\text{2}}}[\sum_{\alpha} \left( {{|p}_{\alpha}|}^{\text{2}}+\frac{{{|m}_{\alpha}|}^{\text{2}}}{c} \right)+\frac{\text{1}}{\text{120}}\sum_{\alpha} \left( {{|kQ}_{\alpha\beta}^{e}|}^{\text{2}}+\left| \frac{{kQ}_{\alpha\beta}^{e}}{c} \right|^{\text{2}} \right)+\cdots]$ (6)

**
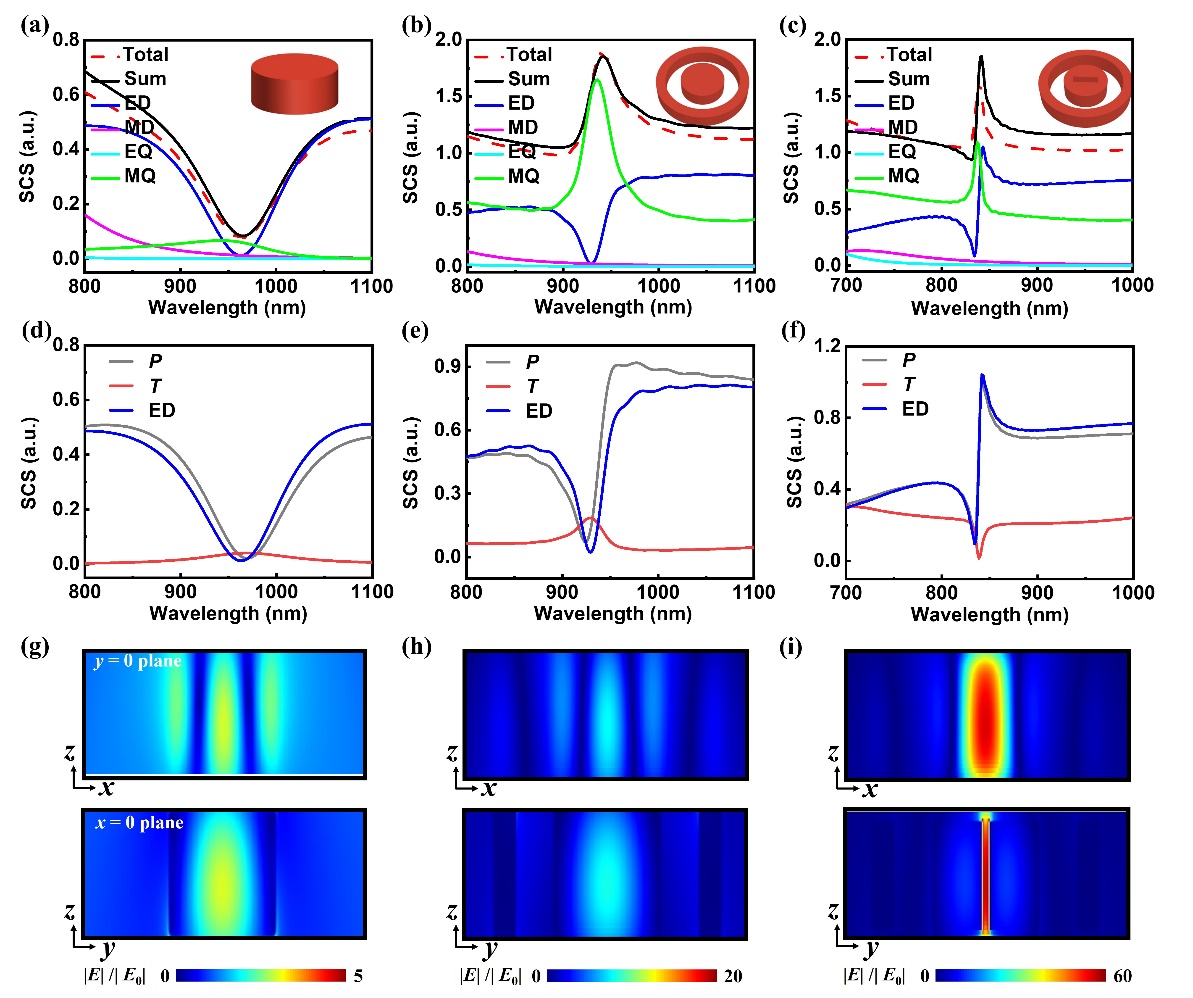
**

**Figure N2**. Optical responses of individual Si disk (SD), solid Si DR (SDR) and slotted Si DR (SSDR) meta-structures. (a-c) The contributions from multipole modes to the scattering spectra of the SD (a), SDR (b) and SSDR (c), respectively. They are ED (blue line), MD (magenta line), EQ (cyan line), MQ (green line) and total contribution (Sum, black line) as well as the simulation calculation (Total, red dashed line). (d-f) The contribution of the original electric dipole (***P***, gray line) mode, toroidal dipole (***T***, red line) mode, and ED (blue line) mode to scattering field. (g-i) The near-field profiles on the *y* = 0 plane (top) and *x* = 0 (bottom) plane of the SD (g), SDR (h) and SSDR (i) at resonant wavelengths, respectively.

Here, the contributions of the multipole modes are presented. Through comparative analysis, it can be intuitively found that no matter far field response or near field effect, the SSDR meta-structure has overwhelming advantages such large EF and low radiation loss. The red dashed line in Figures N2a-c denotes scattering spectrum of three meta-structures through simulation calculation. Furthermore, the Cartesian multipole contributions based on analytical calculation are also shown in Figure N2a-c, including electric dipole (ED, blue line), magnetic dipole (MD, magenta line), electric quadrupole (EQ, cyan line), magnetic quadrupole (MQ, green line) and the sum of four components (Sum, black line). The subtle deviation of sum and total is attributed to other neglected multipole modes and approximate analytical calculations of the SCS. Herein, the ED contribution already includes the common ED and toroidal dipole (TD) terms,^[11,12]^ showing the interaction of co-excited ED and TD moments with opposite phase, namely the anapole mode. Specifically, Cartesian multipole decomposition results of the SCS for the SD, the SDR and the SSDR nanocavities including original electric dipole (***P***, $p_{\alpha}=\frac{\text{1}}{i\omega}\int d^{\text{3}}\hat{r}J_{\alpha}^{\omega}$, black line)^[13]^, toroidal dipole (***T***, $T_{\alpha}=\frac{\text{1}}{10c}\int d^{\text{3}}\hat{r}[\left( \hat{r}\cdot\hat{J}_{\omega} \right)r_{\alpha}-{2r}^{\text{2}}J_{\alpha}^{\omega}]$, red line)^[14]^ and mode coupled by ***P*** and ***T*** (ED, blue line)^[12]^. When ***P*** and ***T*** have the same amplitude and opposite phase (i.e., ***P*** = ik***T***), there is the destructive interference between ***P*** and ***T***, resulting in the anapole mode as shown in Figure N2d-f. One can observe that the contribution of MD and EQ is almost negligible, which is confirmed by the near-field profiles in the Figure 2c in the main text.

For SD and SDR meta-structures, the EF enhancement within the nonlinear interaction region is mid. As shown in Figures N2g-i, we plot EF distributions on the *y* = 0 (top) and *x* = 0 (bottom) planes of three meta-structures at resonant wavelengths, respectively. It is not difficult to find that in the same spatial position, The SSDR has a stronger ability to localize the optical field, resulting in greater EF enhancement.

**Table S1. THG conversion efficiency of the all-dielectric structures.**

| **Reference** | **Nanosystem** | **THG**  **wavelength (nm)** | **THG conversion efficiency *P*_3ω_/*P*_ω_** | **Enhancement factor (THG_Si_/THG_film_)** | **Structural thickness**  **(nm)** | **Pump power**  **(mW)** | **Peak power density (GW/cm^2^)** |
| --- | --- | --- | --- | --- | --- | --- | --- |
| *Nano Lett.* **2014***, 14,* 6488 | Isolated silicon disk e | 420 | 1🞨10^-7^ | 100 | 260 | 30 | 5 |
| *ACS Photon.* **2015***, 2, 578* | silicon nanodisk trimers | 394 | 1🞨10^-10^ | 2.8 | 260 | - | 8 |
| *Philos. Trans. A Math. Phys Eng. Sci.* **2017***, 375,* 20160281 | Isolated silicon disk | 300 | 4🞨10^-8^ | 1.4 | 260 | ~ 40 | - |
| *ACS Photon.* **2018***, 5,* 728 | Isolated silicon disk | 517 | 4🞨10^-8^ | - | 700 | 1.5 | 10 |
| *ACS Photon.* **2018***, 5,* 1671 | Silicon metasurfaces | 427 | 1,76🞨10^-7^ | 220 | 200 | 26.7 | - |
| *ACS Photon.* **2019***, 6,* 1639 | Silicon metasurfaces | 477 | 1🞨10^-6^ | - | 538 | 130 | - |
| *Nano Lett.* **2020***, 20,* 3471 | Oligomers of silicon disks | 313 | 5.5🞨10^-8^ | 120 | 260 | ~ 40 | - |
| *Adv. Funct. Mater.* **2021***, 31,* 2104627 | Hybrid dielectric metasurfaces | 403 | 2.9🞨10^-8^ | 32 | 400 | 55 | 1.6 |
| *Light Sci. Appl.* **2023***, 12,* 97 | Silicon metasurfaces | 517 | 2.8🞨10^-7^ | 900 | 590 | 150 | 1.2 |
| *Nano Lett.* **2024***, 24,* 2257 | Silicon metasurfaces | 440 | 3.25🞨10^-5^ | 240 | 695 | - | 23.8 |
| *ACS Nano* **2024***, 18,* 4388 | Silicon metasurfaces | 280 | 5.2🞨10^-8^ | 16 | 400 | 280 | 15 |
| **Our work** | **Isolated silicon disk-ring** | **309** | **6.12🞨10^-5^** | **117** | **100** | **50** | **13.3** |

**Reference**

[1] E. V. Melik-Gaykazyan, M. R. Shcherbakov, A. S. Shorokhov, I. Staude, I. Brener, D. N. Neshev, Y. S. Kivshar, A. A. Fedyanin, *Philos. Trans. A Math. Phys Eng. Sci.* **2017**, *375*, 20160281.

[2] O. A. M. Abdelraouf, A. P. Anthur, X. R. Wang, Q. J. Wang, H. Liu, *ACS Nano* **2024**, *18*, 4388.

[3] R. Xie, X. He, W. Wang, L. Zheng, J. Shi, *Photonics* **2024**, *11*, 159.

[4] N. K. Hon, R. Soref, B. Jalali, *J. Appl. Phys.* **2011**, *110*, 011301.

[5] A.-Y. Liu, J.-C. Hsieh, K.-I. Lin, S. H. Tseng, H.-H. Hsiao, *Adv. Opt. Mater.* **2023**, *11*, 2300526.

[6] K. Du, P. Li, K. Gao, H. Wang, Z. Yang, W. Zhang, F. Xiao, S. J. Chua, T. Mei, *J. Phys. Chem. Lett.* **2019**, *10*, 4699.

[7] D. E. Aspnes, A. A. Studna, *Phys. Rev. B* **1983**, *27*, 985.

[8] A. E. Miroshnichenko, A. B. Evlyukhin, Y. F. Yu, R. M. Bakker, A. Chipouline, A. I. Kuznetsov, B. Luk'yanchuk, B. N. Chichkov, Y. S. Kivshar, *Nat. Commun.* **2015**, *6*, 8069.

[9] N. Papasimakis, V. A. Fedotov, V. Savinov, T. A. Raybould, N. I. Zheludev, *Nat. Mater.* **2016**, *15*, 263.

[10] E. E. Radescu, G. Vaman, *Phys. Rev. E* **2002**, *65*, 046609.

[11] R. Alaee, C. Rockstuhl, I. J. O. C. Fernandez-Corbaton, *Opt. Commun.* **2018**, *407*, 17.

[12] Y.-H. Deng, Z.-J. Yang, M.-L. Hu, X.-J. Du, J. He, *New J. Phys.* **2021**, *23*, 023004.

[13] Z.-J. Yang, Q. Zhao, J. He, *J. Appl. Phys.* **2019**, *125*, 063103.

[14] J. Wang, W. Yang, G. Sun, Y. He, P. Ren, Z. Yang, *Photon. Res.* **2022**, *10*, 1744.
